# Assessing the impact of a second IVIG dose on patients with Guillain–Barré Syndrome: A synthesis without meta-analysis (SWiM)

**DOI:** 10.5339/qmj.2025.88

**Published:** 2025-09-25

**Authors:** Mohammad Minwer Alnaeem, Ahmad R. Al-Qudimat, Doaa Talafha, Omar Alqaisi

**Affiliations:** 1 AI-Zaytoonah University of Jordan, School of Nursing, Amman, Jordan; 2 Surgical Research Section, Surgery Department, Hamad Medical Corporation, Doha, Qatar; 3 Public Health Department, Health Sciences College, Qatar University, Doha, Qatar *Email: aalqudimat@hamad.qa

**Keywords:** Guillain–Barré syndrome, IVIG, prognosis, second dose, treatment

## Abstract

**Background and Aim:**

Guillain–Barré Syndrome (GBS) is a severe neurological disorder in which the immune system attacks the peripheral nervous system, leading to acute flaccid paralysis. The conventional treatment involves intravenous immunoglobulin (IVIG), yet the efficacy of a second IVIG dose remains uncertain. This systematic review aims to evaluate the clinical outcomes of administering a second dose of IVIG in GBS patients.

**Methods:**

The review was registered in the PROSPERO database (CRD42024557465) and adhered to Preferred Reporting Items for Systematic Reviews and Meta-Analyses (PRISMA) standards. Databases including EBSCO, MEDLINE, CINAHL, and SCOPUS were searched from database inception until June 2024, using a combination of relevant keywords. Only English-language studies were included. Quality assessment was conducted using the Joanna Briggs Institute critical appraisal tools. In total, 89 abstracts were initially screened, 20 full-text articles were reviewed, and 10 studies met the inclusion criteria for final evaluation.

**Results:**

The review included 10 studies with 94 patients across all included studies (31 female, 63 male). This review includes two clinical trials, six case studies, and two case report series, conducted across Europe and Asia. The studies evaluated the clinical outcomes of a second IVIG dose in GBS patients in various healthcare settings, such as emergency rooms, medical departments, and intensive care units. Most of these studies reported significant clinical improvements in motor symptoms and successful weaning from mechanical ventilation following the administration of a second IVIG dose. Eighty percent of the studies included patients on mechanical ventilation, with a standard IVIG dose of 0.4 g/kg reported in 60% of studies. Half of the studies administered the second IVIG dose 2 weeks after the first. Seventy percent of the studies reported clinical motor improvement following the second IVIG dose, with half indicating successful weaning from mechanical ventilation. Evaluation methods varied, with cerebrospinal fluid protein testing (60%) and nerve conduction studies (50%) being the most common. A smaller proportion of studies assessed GMI/GDIA antibodies, immunoglobulin G (IgG) levels, or conducted electrophysiological studies. The findings demonstrate the potential effectiveness of a second IVIG dose in improving motor outcomes and reducing ventilator dependency in GBS patients.

**Conclusion:**

The findings suggest that a second dose of IVIG may enhance clinical outcomes in GBS patients, particularly in improving motor functions and facilitating weaning from mechanical ventilation. However, further prospective randomized trials are essential to validate these results and improve treatment protocols.

## 1. INTRODUCTION

Guillain–Barré Syndrome (GBS) is a medical condition in which the immune system attacks the peripheral nervous system, causing issues with muscles and nerves that control sensations such as touch, pain, and temperature.^1^ This condition can also lead to difficulties with breathing and swallowing. A study conducted by Walgaard et al.^2^ indicates that this illness can cause sudden limb weakness and is typically a one-time occurrence.

The incidence of GBS varies from 0.59 to 2.35 cases per 100,000 individuals globally. The fatality rate ranges from 3% to 13%,^3,4^ highlighting its severity. Common complications include respiratory, cardiovascular, and vascular issues. GBS often presents with a spectrum of sensory and autonomic disturbances, including tachycardia, blood pressure fluctuations, and orthostatic hypotension, which can be clinically alarming.^5^ Owing to the potentially severe nature of these symptoms, most patients require hospitalization and close clinical monitoring to reduce the risk of morbidity and mortality.^5,6^ GBS typically arises within 4 to 6 weeks following an immune-triggering event, most often gastrointestinal or respiratory infections.^7–9^ Among infectious agents, *Campylobacter jejuni* is the most commonly implicated pathogen in Western countries, followed by cytomegalovirus, Epstein-Barr virus, and influenza A.^10^ In rare cases, trauma or vaccinations may also serve as triggers.^11^ Although the exact pathogenesis of GBS is not completely understood, molecular mimicry is believed to play an important role. In this mechanism, gangliosides have structural similarities with microbial antigens, leading the immune system to erroneously attack the peripheral nerves, resulting in varying degrees of paralysis.^12^

While the diagnosis of GBS is primarily based on clinical indicators, additional diagnostic tests are available, such as elevated protein levels in the cerebrospinal fluid (CSF), antiganglioside antibodies, and electrodiagnostic assessments. Intravenous immunoglobulin (IVIG) and plasma exchange are effective treatments for GBS.^2^ However, despite these treatments, approximately 20% of patients are unable to walk after 6 months, and 20%–30% require mechanical ventilation.^2^ A standard course of IVIG involves a dose of 2 g/kg given over 2–5 days. Administered within the first 2 weeks, IVIG can shorten the recovery time from GBS.^13^ Patients with a poor early prognosis may require additional treatment. Despite limited evidence supporting this approach, administering a second dose of IVIG in the early stages, before severe or irreversible nerve damage occurs, may be beneficial.^2^

Several studies have suggested that the first dose of IVIG provides only marginal improvement.^13^ Although a few patients have reported better symptoms after receiving a second dose, evidence remains limited. As a result, a systematic review was conducted to determine whether administering a second dose of IVIG leads to better outcomes.

## 2. METHODS

This systematic review has been conducted following the Preferred Reporting Items for Systematic Reviews and Meta-Analyses (PRISMA) and Synthesis Without Meta-Analysis (SWiM) guidelines.^14^

### 2.1 Data source and search strategy

**Figure 1:**
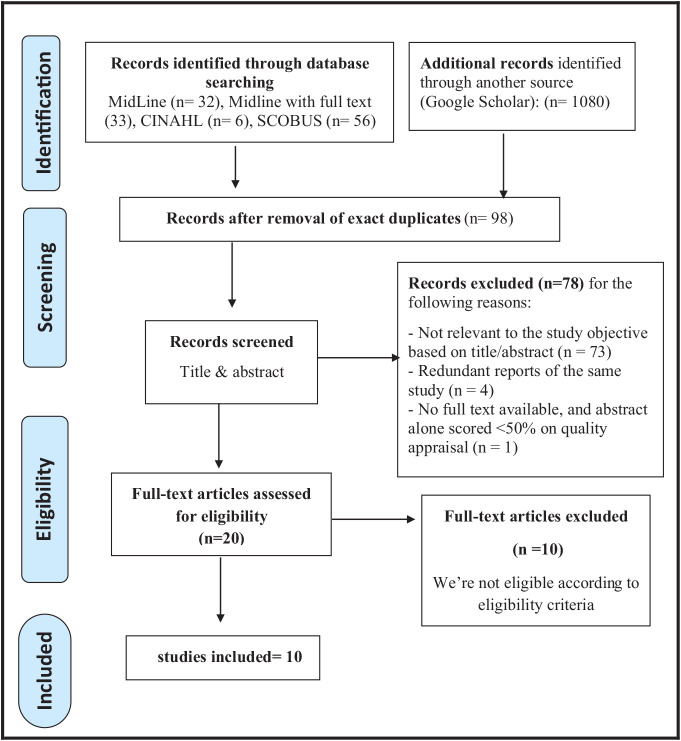
PRISMA flowchart (flow diagram of the systematic search strategy).

This review was registered in the PROSPERO database (CRD42024557465) for systematic reviews. Researchers conducted an intensive search using EBSCO, MEDLINE, CINAHL, Google Scholar, and SCOPUS databases. The search strategy was meticulously based on the PRISMA standards for systematic reviews, ensuring the highest quality of research, to examine the effectiveness of a second IVIG dose in GBS. Articles were searched from database inception up until June 2024, with no restrictions on the publication date. A combination of keywords was used, including ((“intravenous IVIG” OR “immunoglobulin”) AND (“Guillain-Barre syndrome” OR “GBS”) AND (“second intravenous immunoglobulin” OR “second IVIG”)). Although subtypes such as acute inflammatory demyelinating polyradiculoneuropathy (AIDP) were not explicitly included in the search terms, studies categorized under the broader GBS terminology were included to ensure comprehensive coverage. Only English language studies published up until June 2024 were included. All non-English articles, review articles, abstracts, and non-peer-reviewed sources were excluded. A total of 98 records were identified after removing exact duplicates. These were screened based on titles and abstracts. A total of 78 records were excluded for the following reasons: the study’s objective was not relevant based on the title or abstract (*n* = 73), redundant reports of the same study (*n* = 4), and one study was excluded due to the unavailability of full text and a critical appraisal score below 50% based on the abstract alone (*n* = 1), making it insufficient for inclusion. The remaining 20 full-text articles were assessed for eligibility, and 10 met the inclusion criteria for the final review (Figure 1).

### 2.2 Study selection and eligibility

The inclusion criteria were based on the PICO model. The population (P) consisted of patients diagnosed with GBS, validated by clinical or electrophysiological criteria. The intervention (I) involved receiving a second cycle of the standard dose of IVIG. The outcomes (O) included the dose and timing of administering the second IVIG dose, clinical improvements, GBS complications, and tests confirming improvement. The timeframe (T) spanned from database inception up to June 2024. Studies conducted among patients who received a high dose or only a standard single dose of IVIG were excluded.

### 2.3 Data extraction and synthesis

All relevant studies were selected based on inclusion and exclusion criteria. Two reviewers independently screened the titles and abstracts of all identified articles. When titles or abstracts did not provide sufficient detail, or disagreements among researchers arose, the full text (when available or requested) was reviewed to determine the paper’s eligibility. Any disagreements between reviewers were resolved through consensus or with input from a third reviewer.

Data were extracted using a standardized form that included study title, publication year, study design, sample size, setting, and main outcomes (Table 1). According to PRISMA, we specifically sought outcome data related to (1) the timing and dose of the second IVIG cycle, (2) clinical improvements (especially in motor function), (3) complications such as respiratory failure or ventilator dependence, and (4) diagnostic tests supporting clinical improvement (e.g., CSF protein levels, nerve conduction studies, antibody profiles). Although not all studies reported on every outcome, we extracted all results that were compatible with the predefined domains. When multiple results were presented (e.g., several time points or outcome measures), we prioritized those that reflected early and clinically significant improvements after the second IVIG dose. This method allowed us to compare findings consistently across heterogeneous study designs. Furthermore, due to the heterogeneity in study designs, sample sizes, and outcome measures, a narrative synthesis rather than a meta-analysis was used to summarize and interpret the findings across studies.

### 2.4 Quality assessment of included studies

Given the nature of the included studies (case reports, case series, and small clinical trials), a formal assessment of reporting bias and certainty of evidence was not applicable. However, a comprehensive quality assessment was performed using the Joanna Briggs Institute (JBI) critical appraisal tools to evaluate methodological rigor. A summary of the quality assessment using the JBI tools for the included studies is presented in Table 2. Case reports and case series were evaluated using eight questions, each scored as “1” for “Yes” and “0” for “No,” “Unclear,” or “Not applicable.” The assessment covers eight different elements: patient demographics, medical history, health status, physical examination and diagnosis, concomitant therapies, post-intervention health status, and drug administration reaction interface. Articles achieving scores of ≥ 7, 4–6, or < 4 are considered high, moderate, and low quality, respectively. For experimental studies such as randomized controlled trials (RCTs) or quasi-experimental studies, there are 13 and 9 questions, respectively. The quality of these studies is interpreted based on the total score for each tool: ≥ 80%, 50-70%, and < 50% are considered high, moderate, and low quality, respectively.

## 3. FINDING

### 3.1 Characteristics of included studies

The systematic review included a diverse range of study designs and patient characteristics to evaluate the clinical outcomes of a second IVIG dose in GBS patients. Among the ten studies, two were clinical trials, six were case studies, and two were case report series. The studies were geographically balanced, with five conducted in Europe and five in Asia. The studies included various healthcare settings, including emergency rooms, medical departments, and intensive care units, highlighting the comprehensive nature of the review (Table 2).

### 3.2 Quality of the studies

In the six included case reports, the overall quality score was high. Five studies achieved high-quality scores,^15,17–19,23^ while one study had a moderate quality score^16^ due to limited explanations of the patient’s history, diagnostic tests, and adverse events in the published cases.

For the case series, there were two studies, both of which had high-quality scores of 8^24^ and 9^20^ out of 10. However, both studies failed to report any considerations of statistical analysis. Among the experimental studies, there was an RCT^2^ which achieved a moderate-quality score due to bias related to the administration of the intervention/exposure and bias in the statistical conclusion validity. On the contrary, the quasi-experimental study^22^ achieved a high-quality score (Table 3).

### 3.3 Clinical characteristics of patients in the included studies

The review included 10 studies with a total of 94 patients (31 females, 63 males) who received a second dose of IVIG. The sample sizes and treatment groups varied across studies. For instance, in Verboon et al.,^22^ out of 237 eligible patients, 38 patients received a second IVIG course—20 received an early second dose (1–2 weeks after the first dose) and 18 received a late second dose (2–4 weeks after the first dose). Similarly, in Walgaard et al.,^25^ among 93 patients with a poor prognosis, 49 patients were randomized to receive a second IVIG dose, while 44 received a placebo (Table 4). Most studies (*n* = 8, 80%) reported that the included patients were connected to mechanical ventilators, and the administered dose of IVIG was 0.4 g/kg (*n* = 6, 60%). Half of the studies (*n* = 5, 50%) reported that the second dose of IVIG was administered 2 weeks after the first dose. Seven studies (70%) found that the patients either had no comorbidities (*n* = 4) or did not mention their medical history (*n* = 3).

Most studies (*n* = 7, 70%) reported clinical improvement in motor systems after patients received the second dose of IVIG, and half of the studies (*n* = 5, 50%) indicated that patients were weaned off the ventilator. The evaluation tests used to confirm clinical improvement were mostly CSF tests for protein (*n* = 6, 60%) or nerve conduction studies (*n* = 5, 50%). However, a few studies (20%) examined GMI or GDIA antibodies and IgG levels or performed electrophysiological studies to confirm improvement in the motor system (Table 4).

## 4. DISCUSSION

GBS is a condition where the immune system causes acute damage to peripheral nerves, leading to the production of neurotoxic antibodies. The conventional treatment for GBS is IVIG at high doses; however, the exact mechanism of action is uncertain, and the response to treatment varies.^26^ This review, which aimed to assess the outcomes of administering a second dose of IVIG for GBS patients, provides crucial insights for medical professionals and researchers in the fields of neurology and immunology.

GBS is the most common cause of acute flaccid paralysis worldwide. Most patients experience a preceding illness, typically an upper respiratory tract infection, before the onset of increasing motor weakness. Several microbes have been linked to GBS, including Campylobacter jejuni, Zika virus, and the coronavirus responsible for severe acute respiratory syndrome in 2020.^27^ Our review found that most patients were connected to mechanical ventilators. Twenty to thirty percent of individuals present with a generalized form of GBS associated with respiratory failure.^28–31^ As the disease progresses, respiratory muscle weakness is the leading cause of acute respiratory distress and respiratory failure with hypoxia and/or hypercarbia. Hence, mechanical ventilation is often necessary due to the severity of respiratory failure in GBS patients, highlighting the associated risks and complications. In addition to supportive care, treatment with IVIG or plasma exchange is the best option.^32^ Most studies revealed that GBS patients received a second dose of IVIG of 0.4 g/kg (*n* = 6, 60%). This finding is consistent with several previous studies that found IVIG at 0.4 g/kg/day for five days to be a common and preferred treatment regimen for GBS.^33–36^

Most studies in our review revealed that the second dose of IVIG was administered approximately two weeks after the first dose. Similarly, Verboon et al.^37^ and Verboon et al.^22^ reported the same recommended interval between the first and second doses of IVIG. In our review, most patients showed clinical improvement in their motor symptoms after receiving the second dose of IVIG. Previous studies reported similar conclusions.^13,38,39^ Many reviewed studies found that GBS patients improved and were able to be weaned off the ventilator after receiving the second dose of IVIG. Although it is highly unlikely for patients to be weaned from the ventilator within 3 weeks of intubation, our review found that GBS patients who received the second dose of IVIG were eventually successfully weaned.^40–42^ However, a randomized trial included in our review did not demonstrate that a second IVIG course significantly improved clinical outcomes in patients with GBS.

Notably, pharmacokinetic modeling studies have shown that while a second course of IVIG increases serum IgG levels in accordance with model predictions, it does not necessarily translate into better clinical outcomes.^43–46^ In fact, a higher incidence of serious adverse events, including thromboembolic complications, was observed in the second IVIG group, particularly among those with lower IVIG exposure.^25,47,48^ These findings highlight that individual variability in IgG response could impact clinical effectiveness, emphasizing the potential role of model-informed precision dosing in guiding tailored treatment decisions.^33,44,48^

### 4.1 Clinical implications

The findings of this systematic review have significant clinical implications for the treatment and management of GBS. The administration of a second dose of IVIG appears to offer notable benefits in terms of motor function improvement and the ability to wean patients off mechanical ventilation. Given the high morbidity associated with GBS and the substantial proportion of patients requiring intensive care support, these results suggest a potentially impactful strategy for enhancing patient outcomes. Clinicians should consider the timing and dosage of a second IVIG administration, with evidence suggesting a second dose of 0.4 g/kg administered approximately two weeks after the initial dose may be effective. This approach could be particularly beneficial for patients who do not exhibit adequate recovery following the first IVIG dose. Furthermore, regular monitoring and diagnostic tests, such as CSF protein analysis and nerve conduction studies, should be used to assess clinical improvements and guide treatment decisions.

These findings underscore the need for further prospective randomized trials to confirm the efficacy of a second IVIG dose and to develop standardized treatment protocols. In the interim, healthcare providers might integrate these insights into their practice, potentially improving the quality of care and recovery outcomes for patients with GBS. Adopting this approach could reduce the duration of mechanical ventilation and associated complications, ultimately enhancing the overall prognosis for GBS patients.

As GBS affects the peripheral nervous system, CSF is a potential source for biomarkers, as the CSF compartment is in close contact with the proximal nerve roots, where biochemical changes related to the disease are likely to be reflected.^49^ In our review, the most common diagnostic tests used to confirm clinical improvement were CSF tests for protein and nerve conduction studies (*n* = 5, 50%). This finding is consistent with the previous literature.^49,50^ However, other studies highlighted the role of specific antibodies and proteins in the diagnosis and prognosis of GBS, emphasizing the importance of these biomarkers in understanding the disease.^51–53^ In our review, a few studies (20%) examined GMI or GDIA antibodies and IgG levels or performed electrophysiological studies. Furthermore, our review revealed that the main clinical manifestations were respiratory failure, limb weakness, paresthesia, and quadriplegia. Most patients either had no comorbidities or did not mention their medical history. In contrast, previous studies found that patients with GBS mostly presented with a medical history or comorbidities.^31,54–56^ It is important to note that GBS incidence increases by 20% for every 10-year increase in age.^57^ Finally, it is crucial to consider conducting a prospective randomized trial to determine whether a second IVIG therapy enhances GBS outcomes, despite the uncertainty about its benefits. This call for further research should inspire healthcare professionals and researchers to continue their efforts in understanding and treating GBS.

### 4.2 Limitation

This study was limited by the small sample sizes and the severe condition of the patients in many of the included studies, which may have been related to advanced axonal damage occurring before the second IVIG course was administered. In addition, the heterogeneity in the outcome measures and assessment methods across studies reduced the comparability of results. Many studies also lacked control groups, which makes it challenging to isolate the effect of the second IVIG dose from other potential interventions. Another limitation was the exclusion of studies focused exclusively on AIDP, as specific terms for this subtype were not included in the search strategy, potentially reducing the comprehensiveness of the literature review. Furthermore, the retrospective designs of several included studies may have introduced selection and recall biases, impacting the generalizability of the findings.

## 5. CONCLUSION

This systematic review highlights the potential benefits of administering a second dose of IVIG in patients with GBS. While several case reports and small studies suggest potential clinical improvements following the second IVIG dose, such as better motor function and successful weaning from mechanical ventilation, these findings must be interpreted with caution due to the limited number of high-quality studies. Although some diagnostic assessments, such as CSF protein levels and nerve conduction studies, supported observed improvements, the current evidence remains insufficient to draw definitive conclusions. Therefore, a second IVIG dose should be considered only on a case-by-case basis until more robust evidence becomes available. Further prospective randomized controlled trials are necessary to validate these findings and optimize treatment protocols.

## DATA AVAILABILITY STATEMENT

All data analyzed during this study are included in this article, and further inquiries can be directed to the corresponding author.

## AUTHORS’ CONTRIBUTION

MMA, DT, and OA performed the literature search and collected and interpreted the data. MMA, DT, OA, and ARA-Q drafted the work and contributed to the writing of this manuscript. MMA and ARA-Q edited and drafted the final version of this manuscript. All authors reviewed the final version to be published.

## ACKNOWLEDGMENT

None.

## COMPETING INTERESTS

The authors declare that they have no conflict of interest.

## INFORMED CONSENT

Not applicable.

## Figures and Tables

**Table 1.** Findings of the review.

		No. of studies (%)	Reference
**Dose of IVIG**			
• 0.4 g/kg		6 (60%)	Lopes et al.;^15^ Vembu et al.;^16^ Miyamoto et al.;^17^ Shalman et al.^18^ Okoh et al.;^19^ Godoy and Rabinstein^20^
• 2 g/kg		3 (30%)	Farcas et al.;^21^ Walgaard et al.^2^ Verboon et al.^22^
**Time of administration of second dose of IVIG**			
• After 2 weeks from first dose		5 (50%)	Verboon et al.;^22^ Farcas et al.^21^ Shalman et al.;^18^ Vembu et al.^16^ Lopes et al.^15^
• After 4 weeks		2 (20%)	Verboon et al.;^22^ Miyamoto et al.^17^
• More than 8 weeks		3 (30%)	Walgaard et al.;^2^ Godoy and Rabinstein;^20^ Okoh et al.^19^
**Medical device**			
• Mechanical ventilator		8 (80%)	Walgaard et al.;^2^ Verboon et al.^22^ Farcas et al.;^21^ Godoy and Rabinstein;^20^ Okoh et al.;^19^ Shalman et al.;^18^ Miyamoto et al.;^17^ Kinboshi et al.^23^
• Other type of treatment or intervention with IVIG		4 (40%)	Lopes et al.;^15^ Vembu et al.^16^ Miyamoto et al.;^17^ Okoh et al.^19^
Yes		3 (30%)	Lopes et al.;^15^ Vembu et al.^16^ Godoy and Rabinstein^20^
**Comorbidity**			
No		4 (40%)	Shalman et al.;^18^ Miyamoto et al.^17^ Kinboshi et al.;^23^ Okoh et al.^19^
Not clear		3 (30%)	Farcas et al.;^21^ Walgaard et al.^2^ Verboon et al.^22^
**Main outcome**			
• No better outcomes after a second IVIG course		2 (20%)	Verboon et al.;^22^ Walgaard et al.^2^
• Clinical improvement (motor function)		7 (70%)	Vembu et al.;^16^ Lopes et al.^15^ Miyamoto et al.;^17^ Shalman et al.^20^ Okoh et al.;^19^ Godoy and Rabinstein;^20^ Farcas et al.^21^
• Weaning from mechanical ventilation		5 (50%)	Kinboshi et al.;^23^ Miyamoto et al.^17^ Shalman et al.;^18^ Okoh et al.^19^ Godoy and Rabinstein^20^
**Test**			
• Cerebrospinal fluid	proteins (mg/dl)	6 (60%)	Farcas et al.;^21^ Lopes et al.^15^ Vembu et al.;^16^ Shalman et al.^18^ Godoy and Rabinstein;^20^ Okoh et al.^19^
	cells/mm^3^	4	Okoh et al.;^19^ Godoy and Rabinstein;^20^ Vembu et al.;^16^ Lopes et al.^15^
• Electrophysiological studies		2 (20%)	Farcas et al.;^21^ Verboon et al.^22^
• Nerve conduction studies		5 (50%)	Walgaard et al.;^2^ Verboon et al^22^ Okoh et al.;^19^ Vembu et al.^16^ Kinboshi et al.^23^
• GM1 or GD1a antibodies		2 (20%)	Walgaard et al.;^2^ Farcas et al.^21^
• IgG level		2 (20%)	Walgaard et al.;^2^ Verboon et al.^22^
• Motor exam (scale/5) or muscle strength		4 (4%)	Lopes et al.;^15^ Miyamoto et al.^17^ Shalman et al.^18^; Okoh et al.^19^

**Table 2.** Characteristics of literature included in the review (*N* = 10).

Characteristics	No. of studies (%)	Author name (year)
**Study design**		
Clinical trials	2 (20%)	Walgaard et al.;^2^ Verboon et al.;^22^
Case study	6 (60%)	Lopes et al.;^15^ Kinboshi et al.;^23^ Vembu et al.;^16^ Miyamoto et al.;^17^ Shalman et al.;^18^ Okoh et al.^19^
Case report series	2 (20%)	Godoy and Rabinstein;^20^ Farcas et al.^21^
**Gender**		
No. of female patients	31 (*n* = 7)	Vembu et al.;^16^ Godoy and Rabinstein;^20^ Kinboshi et al.;^23^ Okoh et al.;^19^ Verboon et al.;^22^ Walgaard et al.;^2^ Farcas et al.^21^
No. of male patients	63 (*n* = 7)	Shalman et al.;^18^ Miyamoto et al.;^17^ Godoy and Rabinstein;^20^ Lopes et al.;^15^ Verboon et al.;^22^ Walgaard et al.;^2^ Farcas et al.^21^
**Age group**		
Less than 40 years	3	Shalman et al.;^18^ Vembu et al.;^16^ Miyamoto et al.^17^
More than 40 years	6	Godoy and Rabinstein;^20^ Kinboshi et al.;^23^ Okoh et al.;^19^ Lopes et al.;^15^ Verboon et al.;^22^ Walgaard et al.^2^
Not clear	1	Farcas et al.^21^
**Country**		
Countries in Europe	5	Walgaard et al.;^2^ Verboon et al.;^22^ Godoy and Rabinstein;^20^ Okoh et al.;^19^ Lopes et al.;^15^
Countries in Asia	5	Shalman et al.;^18^ Farcas et al.;^21^ Kinboshi et al.;^23^ Miyamoto et al.;^17^ Vembu et al.^16^
**Department of care**		
Emergency room	1	Okoh et al.^19^
Medical departments	4	Kinboshi et al.;^23^ Vembu et al.;^16^ Lopes et al.;^15^ Farcas et al.^21^
Intensive care unit	3	Shalman et al.;^18^ Godoy and Rabinstein;^20^ Miyamoto et al.^17^
Not clear	2	Walgaard et al.;^2^ Verboon et al.^22^
**Publication years**		
1995–2005	2	Farcas et al.;^21^ Vembu et al.^16^
2006–2015	2	Okoh et al.;^19^ Godoy and Rabinstein^20^
2016–2024	6	Walgaard et al.;^2^ Verboon et al.;^22^ Lopes et al.;^15^ Kinboshi et al.;^23^ Miyamoto et al.;^17^ Shalman et al.^18^

**Table 3.** JBI critical appraisal tool for case reports.

**(JBI) Critical appraisal checklist for randomized controlled trials**														
Study	Q1	Q2	Q3	Q4	Q5	Q6	Q7	Q8	Q9	Q10	Q11	Q12	Q13	Total %
Walgaard et al.^2^	Y	Y	Y	UN	NA	N	Y	Y	Y	Y	UN	Y	UN	62%, moderate
**JBI critical appraisal checklist for quasi-experimental studies (non-randomized experimental studies)**														
Study	Q1	Q2	Q3	Q4	Q5	Q6	Q7	Q8	Q9					Total %
Verboon et al.^22^	Y	Y	N	Y	Y	Y	Y	Y	Y					89%, high
**Critical appraisal checklist for case series**														
Study	Q1	Q2	Q3	Q4	Q5	Q6	Q7	Q8	Q9	Q10				Total %
Farcas et al.^24^	Y	Y	Y	Y	Y	Y	Y	Y	UN	NA				8/10, high
Godoy and Rabinstein^20^	Y	Y	Y	Y	Y	Y	Y	Y	Y	NA				9/10, high
**Critical appraisal checklist for case reports**														
Study	Q1	Q2	Q3	Q4	Q5	Q6	Q7	Q8						Total
Okoh et al.^19^	Y	Y	Y	Y	Y	Y	Y	Y						8/8, high
Shalman et al.^18^	Y	Y	Y	Y	Y	Y	UN	Y						7/8, high
Miyamoto et al.^17^	Y	Y	Y	Y	Y	Y	UN	Y						7/8, high
Vembu et al.^16^	Y	UN	Y	X	Y	Y	UN	Y						5/8, moderate
Kinboshi et al.^23^	Y	Y	Y	Y	Y	Y	UN	Y						7/8, high
Lopes et al.^15^	Y	Y	Y	Y	Y	Y	UN	Y						7/8, high

**Table 4.** Summary of the reviewed studies.

Study title (year)	Study design	Sample	Setting	Results	Conclusion
Lopes et al.^15^	Case report	52-year-old man with HIV, GBS	Portugal Infectious diseases department	The second regimen of IVIG was administered for 5 days (14 days after admission) with dexamethasone 15 mg/day. - Neurological signs stabilized. - Laboratory reassessment showed an elevation of CD4 lymphocyte counts (460/mm^3^ representing 31.5%) and a decrease in HIV-RNA to an undetectable level. - On discharge, presenting a grade 3/5 on the right lower limb, grade 4-/5 on the left lower limb, and a full recovery of upper limb strength.	Muscle strength improved after three weeks after the second regimen of IVIG plus corticosteroids was administered. Screening for HIV in a patient with AIDP may provide a better outcome because of the early start of ART with good central nervous system penetration in HIV-infected patients.
Kinboshi et al.^23^	Case report	85-year-old Japanese female with GBS	Japan Department of Neurology & Rheumatology	Second IVIG course led to the gradual improvement of quadriparesis and bulbar palsy.	The levels of anti-GT1b antibodies were elevated, which is relatively rare in GBS. It was suggested that anti-GT1b antibodies may be related to the development of axonal GBS with bulbar palsy.
Vembu et al.^16^	Case report	32-year-old female patient with acute lymphoblastic leukemia progressed to GBS	Kuwait Department of Neurology & Rheumatology	After the second IVIG improved the patient’s condition slowly and steadily over 12–16 weeks; the patient was able to walk with minimal support.	IVIG was an effective and non-invasive treatment for Guillain–Barré syndrome associated with the malignancy.
Miyamoto et al.^17^	Case report	Case of a 12-year-old boy with GBS	Japan ICU	- After continued rehabilitation, he was able to walk long distances (functional grade: 3, 2, and 0 on days 56, 66, and 74, respectively). - Discharged on day 89	In cases of severe GBS, when IVIG and pulse steroid therapy do not improve the respiratory muscle strength of the patient, early tracheostomy may improve the patient’s quality of life during artificial respiration management.
Shalman et al.^18^	Case report	21-year-old male	Israel ICU	After a second 5-day course of IVIG. The albumin rise was accompanied by clinical improvement. The patient regained sensations in his toes and fingers. On the seventh day after initiation of the second course, he was able to move his fingers. Subsequently, he was weaned from mechanical ventilation within a few days.	- No clinical improvement in our patient’s condition, following the first IVIG course - With the second 5-day course of IVIG, an increase in the patient’s albumin level was recorded. - Rise in albumin level after the second course of treatment occurred ahead of the clinical improvement. - Serum albumin levels may be considered a biomarker as part of the decision algorithm.
Okoh et al.^19^	Case report	1 female patient	USA ER/Rehabilitation unit	The patient completed recovery after the second 5-day course of IVIG.	A 41-year-old female who progressed to flaccid paralysis with no neurological improvement with initial immunosuppressive therapy, plasmapheresis, and the first cycle of intravenous (IVIG) but with remarkable and complete recovery after the second 5-day course of IVIG.
Godoy and Rabinstein^20^	Case report series	3 severe cases; with GBS, all were axonal forms, two motor (AMAN) and another sensory-motor (AMSAN)	Argentina & USA Neuro ICU	After the second cycle of IVIG (between 3 and 5 days post-infusion), all patients could be weaned from mechanical ventilation. - All became hemodynamically more stable. - Fluctuations in heart rate, blood pressure, and profuse sweating episodes disappeared. - Cardiac rhythm disorders (SVT in two individuals and five episodes of ventricular arrhythmias in two patients) were normalized. - Two patients regained safe swallowing, and another required a feeding tube for 3 more weeks. - At 6 months, none of the 3 was able to walk without assistance and one remained tracheostomized for management of secretions.	A second cycle of IVIG may be an option for treating severe forms of GBS.
Farcas et al.^21^	Retrospective case series.	12 patients with GBS	ICU	- In eight patients, the condition stabilized and improvement was noted within 10 days after the first dose of IVIG - The last four patients were retreated with IVIG after 14–21 days. - Rapid improvement was noted within days. - All four patients began to walk within several weeks.	- The second course of IVIG had a favorable impact on the clinical course of these patients. - The outcome in these patients suggests that in some GBS cases, the standard dose of IVIG is not sufficient and that, in such cases, it is justified to repeat the treatment.
Walgaard et al.^2^	Randomized, double-blind	Patients (≥12 years) with GBS Patients were enrolled on the initial day of standard intravenous immunoglobulin treatment, receiving 2 g/kg over a span of 5 days. Only those with a poor prognosis, as indicated by a score of ≥6 on the modified Erasmus Guillain–Barré Syndrome Outcome Score, were randomly assigned—using block randomization stratified by center—to either receive SID (2 g/kg over 5 days) or a placebo, 7 to 9 days following their inclusion. In a modified intention-to-treat analysis, a total of 93 patients with a poor prognosis were evaluated: 49 (53%) were administered SID, while 44 (47%) received a placebo.	Amsterdam Sitting of care NA	The adjusted common odds ratio for improvement in the Guillain–Barré syndrome disability score at 4 weeks was 1·4 (95% CI = 0·6–3·3; p = 0·45). Patients given SID had more serious adverse events (35% vs 16% in the first 30 days), including thromboembolic events, than those in the placebo group. Four patients died in the intervention group (13–24 weeks after randomization).	We found no significant clinical benefit of a second intravenous immunoglobulin course administered shortly after the first standard intravenous immunoglobulin dose in patients with Guillain–Barré syndrome with poor prognosis.
Verboon et al.^22^	Prospective, observational cohort study	Of 237 eligible patients, 199 patients received a single IVIG course. Twenty patients received an “early” second IVIG course (1-2 weeks after the start of the first IVIG course) and 18 patients a “late” second IVIG course (2-4 weeks after the start of IVIG). Therefore, 38 patients received a second dose	Amsterdam Sitting of Care NA	Compared with the one-course group, the adjusted OR for a better GBS disability score at 4 weeks was 0.70 (95% CI = 0.16 to 3.04) for the early group and 0.66 (95% CI = 0.18 to 2.50) for the late group. The secondary endpoints were not in favor of a second IVIG course.	The study did not show better outcomes after a second IVIG course in GBS with poor prognosis.

## References

[1] World Health Organization: WHO (2016). Guillain–Barré syndrome;.

[2] Walgaard C, Jacobs BC, Lingsma HF, Steyerberg EW, van den Berg B, Doets AY (2021;). Second intravenous immunoglobulin dose in patients with Guillain–Barré syndrome with poor prognosis (SID-GBS): A double-blind, randomised, placebo-controlled trial. Lancet Neurol.

[3] World Health Organization: WHO (2023). Guillain–Barré syndrome;.

[4] Yi S-W, Lee JH, Hong J-M, Choi Y-C, Park HJ (2022). Incidence, disability, and mortality in patients with Guillain–Barré syndrome in Korea: A nationwide population-based study. J Clin Neurol.

[5] Chakraborty T, Kramer CL, Wijdicks EFM, Rabinstein AA (2020). Dysautonomia in Guillain–Barré syndrome: Prevalence, clinical spectrum, and outcomes. Neurocrit Care.

[6] Tewedaj ZD, Huluka DK, Kebede YT, Abebe AT, Hussen MS, Mohammed BD (2024). A retrospective analysis of the clinical profile and factors associated with mortality and poor hospital outcomes in adult Guillain–Barre syndrome patients. Sci Rep.

[7] Dalakas M C (2015). Pathogenesis of immune-mediated neuropathies. Biochim Biophys Acta.

[8] Dalakas MC (2020). Guillain–Barré syndrome: The first documented COVID-19-triggered autoimmune neurologic disease: More to come with myositis in the offing. Neurol Neuroimmunol Neuroinflamm.

[9] Umapathi T (2020). Does COVID-19 cause axonal GBS?. J Clin Neurosci.

[10] Finsterer J (2022). Triggers of Guillain–Barré syndrome: Campylobacter jejuni predominates. Int J Mol Sci.

[11] Huang C, Zhang Y, Deng S, Ren Y, Lu W (2020). Trauma-related Guillain–Barré syndrome: Systematic review of an emerging concept. Front Neurol.

[12] Shastri A, Al Aiyan A, Kishore U, Farrugia ME (2023). Immune-mediated neuropathies: Pathophysiology and management. Int J Mol Sci.

[13] Verboon C, Doets AY, Galassi G, Davidson A, Waheed W, Péréon Y (2019). Current treatment practice of Guillain–Barré syndrome. Neurology.

[14] Campbell M., McKenzie J. E., Sowden A., Katikireddi S. V., Brennan S. E., Ellis S. (2020). Synthesis without meta-analysis (SWiM) in systematic reviews: reporting guideline. Bmj.

[15] Lopes M, Marques P, Silva B, Cruz G, Serra JE, Ferreira E (2021). Guillain–Barré syndrome as the first presentation of human immunodeficiency virus infection. BMC Neurol.

[16] Vembu P, Al-Shubaili A, Al-Khuraibet A, Kreze O, Pandita R (2003). Guillain–Barre syndrome in a case of acute lymphoblastic leukaemia: A case report. Med Princ Pract.

[17] Miyamoto M., Imataka G., Ichikawa G., Saito Y., Kashiwagi T., Kaji Y., Yoshihara S (2020;). Successful treatment of a 12yearold boy with GuillainBarré syndrome requiring tracheostomy due to respiratory muscle paralysis: A case report. Experimental and Therapeutic Medicine.

[18] Shalman A, Savir S, Steen YM, Ovanyan A, Boniel N, Koyfman L (2020). Albumin levels as a biomarker for second Intravenous Immunoglobulin (IVIG) treatment in Guillain–Barre syndrome (GBS). J Clin Neurosci.

[19] Okoh HC, Lubana SS, Langevin S, Sanelli-Russo S, Abrudescu A (2015;). A case of systemic lupus erythematosus presenting as Guillain–Barré syndrome. Case Rep Rheumatol.

[20] Godoy DA, Rabinstein A (2015). Is a second cycle of immunoglobulin justified in axonal forms of Guillain–Barré syndrome?. Arq Neuropsiquiatr.

[21] Farcas P, Avnun L, Frisher S, Herishanu YO, Wirguin I (1997). Efficacy of repeated intravenous immunoglobulin in severe unresponsive Guillain–Barré syndrome. Lancet.

[22] Verboon C, van Den Berg B, Cornblath DR, Venema E, Gorson KC, Lunn MP (2020). Second IVIg course in Guillain–Barré syndrome with poor prognosis: The non-randomised ISID study. J Neurol Neurosurg Psychiatry.

[23] Kinboshi M, Morimoto Y, Yoshida T, Kuzume D, Yamasaki M (2019). An elderly case of Guillain–Barré syndrome with anti-GT1b antibodies. Rinsho Shinkeigaku.

[24] Farcas P., Avnun L., Frisher S., Herishanu Y., Wirguin I. (1997). Efficacy of repeated intravenous immunoglobulin in severe unresponsive Guillain-Barré syndrome. The Lancet.

[25] Walgaard C., Jacobs B. C., Lingsma H. F., Steyerberg E. W., van den Berg B., Doets A. Y. (2021). Second intravenous immunoglobulin dose in patients with Guillain-Barré syndrome with poor prognosis (SID-GBS): a double-blind, randomised, placebo-controlled trial. Lancet Neurol.

[26] Brem M. D., Jacobs B. C., van Rijs W., Fokkink W. J. R., Tio-Gillen A. P., Walgaard (2019). IVI g-induced plasmablasts in patients with Guillain-Barré syndrome. Annals of Clinical and Translational Neurology.

[27] Shahrizaila N., Lehmann H. C., Kuwabara S. ((2021)). Guillain-barré syndrome. The lancet.

[28] Shang P, Zhu M, Baker M, Feng J, Zhou C, Zhang HL (2020). Mechanical ventilation in Guillain–Barré syndrome. Expert Rev Clin Immunol.

[29] Melone MA, Heming N, Meng P, Mompoint D, Aboab J, Clair B (2020). Early mechanical ventilation in patients with Guillain–Barré syndrome at high risk of respiratory failure: A randomized trial. Ann Intensive Care.

[30] van den Berg B, Storm EF, Garssen MJ, Blomkwist-Markens PH, Jacobs BC (2018). Clinical outcome of Guillain–Barré syndrome after prolonged mechanical ventilation. J Neurol Neurosurg Psychiatry.

[31] Wu X, Li C, Zhang B, Shen D, Li T, Liu K (2015). Predictors for mechanical ventilation and short-term prognosis in patients with Guillain–Barré syndrome. Crit Care.

[32] Willison H. J., Jacobs B. C., van Doorn P. A. (2016). Guillain-barre syndrome. The Lancet.

[33] Fokkink W, van Tilburg SJ, de Winter BCM, Sassen SDT, van Doorn PA, Koch BCP (2022). Population pharmacokinetic modelling of intravenous immunoglobulin treatment in patients with Guillain–Barré syndrome. Clin Pharmacokinet.

[34] Lin J, Gao Q, Xiao K, Tian D, Hu W, Han Z (2021). Efficacy of therapies in the treatment of Guillain–Barre syndrome: A network meta-analysis. Medicine (Baltimore).

[35] Khan MH, Islam MK, Rahman S, Rahman AKMF, Mohsin M, Sarker SK (2023). Effectiveness of plasmapheresis and IVIG in the treatment of Guillain–Barre syndrome: A cross-sectional observational study. Bangladesh Crit Care J.

[36] Ram D, Aziz M (2016;). G99 (P) IVIG for Guillain–Barré syndrome: Which regimen should i choose?. Arch Dis Child.

[37] Verboon C, Harbo T, Cornblath DR, Hughes RAC, van Doorn PA, Lunn MP (2021). Intravenous immunoglobulin treatment for mild Guillain–Barré syndrome: An international observational study. J Neurol Neurosurg Psychiatry.

[38] Shalem D, Shemer A, Shovman O, Shoenfeld Y, Kivity S (2018). The efficacy of intravenous immunoglobulin in Guillain–Barré syndrome: The experience of a tertiary medical center. Isr Med Assoc J.

[39] Godoy D. A., Rabinstein A. (2015). Is a second cycle of immunoglobulin justified in axonal forms of Guillain-Barré syndrome?. Arquivos de Neuro-Psiquiatria.

[40] Shang P, Feng J, Wu W, Zhang HL (2021). Intensive care and treatment of severe Guillain–Barré syndrome. Front Pharmacol.

[41] Weiss N. (2021). Should We Assess Diaphragmatic Function During Mechanical Ventilation Weaning in Guillain–Barré Syndrome and Myasthenia Gravis Patients?. Neurocritical Care.

[42] Dugernier J, Bialais E, Reychler G, Vinetti M, Hantson P (2015). Neurally adjusted ventilatory assist during weaning from respiratory support in a case of Guillain–Barre syndrome. Respir Care.

[43] Khdair SI, Al-Khareisha L, Abusara OH, Hammad AM, Khudair A (2025). HLA-class II genes association with multiple sclerosis: An immunogenetic prediction among multiple sclerosis Jordanian patients. PLoS One.

[44] Vlam L, Cats EA, Willemse E, Franssen H, Medic J, Piepers S (2014). Pharmacokinetics of intravenous immunoglobulin in multifocal motor neuropathy. J Neurol Neurosurg Psychiatry.

[45] Zihlif M., Abusara O. H., Al-Qerem W., Al-Ibadah M., Mahafza T. M., Al-Akhras F. M., Mahafza N. T. (2024). CRHR1 polymorphism at rs242941, rs242940, and rs72834580: association of symptoms improvement with intranasal corticosteroids in allergic rhinitis Jordanian patients. Drug Metabolism and Personalized Therapy.

[46] Zihlif M, Imraish A, Al-Rawashdeh B, Qteish A, Husami R, Husami R (2021). The association of IgE levels with ADAM33 genetic polymorphisms among asthmatic patients. J Pers Med.

[47] Kapoor M, Hunt I, Spillane J, Bonnett LJ, Hutton EJ, McFadyen J (2022). IVIg-exposure and thromboembolic event risk: Findings from the UK Biobank. J Neurol Neurosurg Psychiatry.

[48] van Tilburg SJ, Huizinga R, Kuitwaard K, Sassen SDT, Walgaard C, van Doorn PA (2025). If it does not help, it might hurt: Pharmacodynamics of a second IVIg course in Guillain–Barré syndrome. Ann Clin Transl Neurol.

[49] Leonhard SE, Mandarakas MR, Gondim FAA, Bateman K, Ferreira MLB, Cornblath DR (2019). Diagnosis and management of Guillain–Barré syndrome in ten steps. Nat Rev Neurol.

[50] Kamal MM, Habib M, Islam MR, Ghose SK, Uddin KG, Chowdhury AH (2018). Cerebrospinal fluid protein level and nerve conduction study as short-term prognostic marker of Guillain–Barré syndrome. Bangladesh J Neurosci.

[51] Kılıç B, Güngör S, Özgör B (2019;). Clinical, electrophysiological findings and evaluation of prognosis of patients with Guillain–Barré syndrome. Turkish J Pediatr.

[52] Syczewska M, Swiecicka A, Szczerbik E, Kalinowska M, Dunin-Wasowicz D, Łukowicz M (2021). Types of gait deviations in children and adolescents with Guillain–Barre syndrome identified using cluster analysis. Biomed Signal Process Control.

[53] Jawaid W, Sana R, Umer SR, Nisa Q, Butt M, Shahbaz N (2021). Relationship between cerebrospinal fluid protein level and electrophysiologic abnormalities in the acute inflammatory demyelinating polyradiculoneuropathy variant of Guillain–Barré syndrome. Ger Med Sci.

[54] Kim S, Han HJ, Shin HY, Kim S W (2022). Old age and multiple comorbidity are associated with delayed diagnosis of Guillain–Barre syndrome. Sci Rep.

[55] Chang KH, Lyu RK, Lin WT, Huang YT, Lin HS, Chang SH (2019). Gulllain-Barre syndrome after trivalent influenza vaccination in adults. Front Neurol.

[56] Maskin LP, Wilken M, Lucci FR, Wisnivesky JP, Barroso F, Wainsztein N (2024). Risk factors for respiratory failure among hospitalized patients with Guillain–Barré syndrome. Neurología (English Edition).

[57] Sejvar JJ, Baughman AL, Wise M, Morgan OW (2011;). Population incidence of Guillain–Barré syndrome: A systematic review and meta-analysis. Neuroepidemiology.

